# Awareness, Attitude, and Willingness Toward Bleeding Control by Bystanders in Riyadh

**DOI:** 10.7759/cureus.30468

**Published:** 2022-10-19

**Authors:** Amr Y Arkoubi, Sajad A Salati, Alwaleed I Almughira, Abdullah I Abuharb, Khalid A Almutairi, Fahad A Alosaimi, Mohammad Aldayel

**Affiliations:** 1 Department of Plastic Surgery, Imam Mohammad Ibn Saud Islamic University, Riyadh, SAU; 2 Department of Surgery, Unaizah College of Medicine and Medical Sciences, Qassim University, Qassim, SAU; 3 College of Medicine, Imam Mohammad Ibn Saud Islamic University, Riyadh, SAU

**Keywords:** bystander, traffic accidents, first aid training, tourniquet, hemorrhage, bleeding

## Abstract

Background

Hemorrhage after trauma is the second leading cause of death in patients in the prehospital environment, and intervention by bystanders before the arrival of professional rescuers has the potential to save lives in such circumstances. No studies have been conducted in Saudi Arabia till date to assess the knowledge and awareness of bleeding control by bystanders. Hence, this study was conducted with an aim to assess the level of awareness, attitude, and willingness toward bleeding control by bystanders in Riyadh city, the capital of Kingdom of Saudi Arabia (KSA).

Methodology

This is an observational cross-sectional survey design that was conducted from July 2022 to August 2022 using an electronic questionnaire targeting populations who live in Riyadh city. MS Excel 2022 was used for data entry and coding, while SPSS Version 26 (IBM Corp., Armonk, NY) was used for data analysis.

Results

In this study, 585 adults from Riyadh city were recruited. Among the participants, 62.9% of them were between 16 and 26 years of age; 55.4% were males and 90.3% were Saudi Arabian citizens. Of the participants, 76.1% reported that they did not have any experience in participation in bleeding control related activity. Fear of causing more harm to the patients by attempting bleeding control was expressed by 65.1%. In general, 40.2% of the participants have adequate knowledge considering controlling of bleeding in an emergency setting. Higher level of education and having a previous first aid training were associated with better knowledge significantly (p=0.001 and 0.012, respectively).

Conclusion

There is a great need to improve the level of awareness about the role of bystanders in bleeding control and to design community-level activities to popularize this important life-saving skill.

## Introduction

Uncontrolled bleeding is the second leading cause of traumatic prehospital death. Motor vehicle accidents are the prime culprits leading to trauma in many countries including the Kingdom of Saudi Arabia. More than 1.2 million people die each year on world roads, and as many as 50 million get injured [1]. According to World Health Organization (WHO) data published in 2018, the road deaths in the kingdom account for 8.75% of all deaths [2] and the number of road deaths over the past decade has ranged from 17.4 to 24 per 100,000 people. Among the trauma victims who arrive at the emergency department, bleeding is responsible for almost half of deaths during the first 24 hours [3]. Hence, in trauma patients, control of bleeding is an essential intervention [4], and sufficient and adequate bleeding control can increase the survival rate of the patient [5]. It might take only a few minutes to bleed significant volumes, but the arrival of healthcare providers at the accident site and further transportation for definitive care leads to a delay. Controlling the bleeding in the first few minutes at the accident site is hence important [6], and these are the circumstances where bystanders next to the injured is the best person to save the life if they are equipped with the basic skills of bleeding control. It is to achieve on-site bleeding control as in the western countries, awareness campaigns like “Stop the Bleed” have been started at community levels. It is against this backdrop that the present study was conducted in Riyadh city, with an aim to assess the level of awareness, attitude, and willingness towar bleeding control by bystanders. This is the first study conducted to assess the knowledge of public in bleeding control in Saudi Arabia.

## Materials and methods

An observational cross-sectional survey was conducted using an electronic questionnaire targeting the adult population living in Riyadh city. Riyadh is the capital and main financial hub of Saudi Arabia with a population of about eight million [7].

Inclusion criteria

Saudi and non-Saudi residents of Riyadh city, from both the genders and age above 16 years, who agreed to participate in the study were included.

Exclusion criteria

Participants associated with healthcare sector (doctors, nurses, health educators) were excluded to prevent bias in the results, as they were assumed to have acquired greater information during professional training and services about trauma-related issues such as bleeding control.

Proper ethical approval from the Institutional Review Board (IRB) of Imam Mohammad Ibn Saud Islamic University Riyadh was obtained under Project Number 302/2022. Each participant was explained the purpose of the study as per the ethical guidelines of Helsinki and assured of the confidentiality/anonymity of the identities and the use of data for research purposes only. The participants who expressed their willingness to participate in the study were requested to sign an informed consent form.

Calculation of sample size

The sample size was calculated by sample size equation for cross-sectional studies as given below:

Sample size = [Z (1-α/2)^2^ p(1-p)] / d^2^

where Z (1-α/2) = standard normal variate; at 5% type 1 error (p < 0.05), its value as derived from Z-table is equal to 1.96. Since the population of Riyadh city is about 8 million and percentage of people above 16 years is about 5 million (62%), p was taken as 0.62; d = absolute error or precision and value selected was 0.05.

After application of values, minimal required sample to be studied was found to be as follows:

Sample size = [(1.96)^2^ x 0.62(1-0.62)] / (0.05)^2^ = 362

To improve the results and accommodate the non-responders, it had been decided to enroll more than 1.5 times the number of participants as calculated by the above formula.

The study was conducted from July to August 2022. A self-designed anonymous questionnaire comprising closed-ended questions in English and Arabic languages and had three sections. The entire interview was supposed to take a maximum of 10 minutes.

The first section contained demographic data (age, residence, educational qualifications, and nature of job, income). The second section contained assessment of the participants’ knowledge and perception by questioning about any previous experience with bleeding control or participation in any first aid training session that might have talked about bleeding control and by providing certain real-life short scenarios.

Finally, participants were assessed for their willingness to offer help to the trauma victims and their comfort level in that situation. After completion of the questionnaire, all the participants were provided links to written material (Appendix) and YouTube videos (Appendix), related to the concept of “Stop the Bleed” techniques of pressure, pack, and tourniquet. At the end, the participants were asked the following:

a) if they found this activity to be useful

b) if they would like to participate in healthcare related survey in future

Microsoft Office (MS) Excel 2022 was used for data entry and coding, while SPSS version 26 (IBM Corp., Armonk, NY) was used for data analysis. Frequency and percent were used for describing categorical variables, while mean and standard deviation were used for describing ongoing variables.

To assess the level of knowledge of the participants, each correct answer was coded as 1, while wrong answers were coded as 0. For each item, the participants were requested to register only a single most appropriate response. The correct options/answers were decided by the content experts (authors 1 and 2) on the basis of information given in the standard textbooks and literature related to trauma. The sum of the results was calculated, and participants were classified as adequate (able to answer more than 66% of the questions correctly) and inadequate. The chi-square test was used to assess the relationship between demographic factors and level of knowledge. All statements were considered significant if the p-value was lower than 0.05.

## Results

In this study, we were able to collect data from 585 adults in Riyadh city, Saudi Arabia. Among the participants, 62.9% of them were between 16 and 26 years of age, while 18.6% were between 27 and 36 years of age. Moreover, 55.4% of the participants were males and 90.3% were Saudi Arabian citizens. Among the participants, 65.5% reported having university degree as the highest educational level and 24.3% reported having high school education. Considering income, 50.8% of the participants reported having a monthly income of lower than 3,000 SR (Saudi Riyal), 11.1% reported having a monthly income of 3,000-5,000 SR, and 9.6% reported having a monthly income of 10,000-15,000 SR. Moreover, 49.1% of the participants were students, while 19.3% were working as office workers and 7.2% were teachers (Table 1).

**Table 1:** Demographic factors of the participants (N=585)

		N	%
Age (years)	16-26	368	62.9%
	27-36	109	18.6%
	37-46	45	7.7%
	47-56	42	7.2%
	>56	21	3.6%
Sex	Male	324	55.4%
	Female	261	44.6%
Nationality	Saudi	528	90.3%
	Non-Saudi	57	9.7%
Region	Central region	384	65.6%
	Eastern region	50	8.5%
	Western region	95	16.2%
	Southern region	24	4.1%
	Northern region	32	5.5%
Level of education	Primary school	1	0.2%
	Middle school	4	0.7%
	High school	142	24.3%
	University	383	65.5%
	Postgraduate	55	9.4%
Income	<3000	297	50.8%
	3000-5000	65	11.1%
	5000-7000	33	5.6%
	7000-10,000	39	6.7%
	10,000-15,000	56	9.6%
	15,000-20,000	38	6.5%
	20,000-30,000	28	4.8%
	>30,000	29	5.0%
Profession	Student	287	49.1%
	Not working	32	5.5%
	Private sector employee	28	4.8%
	Teacher	42	7.2%
	Office work	113	19.3%
	Trader	14	2.4%
	Housewife	11	1.9%
	Other	58	9.9%

Among the participants in the current study, three-quarters of them reported that they did not have any formal medical training in bleeding control (76.1%); however, 85.5% of them reported that they were aware that bleeding after accident is considered a major cause of preventable death. Moreover, 48.9% of the participants were aware that there is a trend of teaching common people some simple techniques of controlling bleeding at the accident scene, where social media was the main source of information for 46.6% of the participants followed by healthcare related posters in malls/ healthcare facilities (33.1%). Moreover, 55.7% of the participants reported that the use of a tourniquet is considered safe and that 59.5% of them would use tourniquet in real life. Considering confidence using tourniquet in the real life, 45.3% of the participants were somewhat comfortable, 18.5% were comfortable, and 12.1% were very comfortable; however, 32.1% were very uncomfortable considering their response to medical emergencies and 21.9% were uncomfortable (Table 2).

**Table 2:** General awareness of the participants toward bleeding control

		N	%
Do you have any previous first aid training?	No	445	76.1%
	Yes	140	23.9%
Are you aware that bleeding after accident is a major cause of preventable death?	No	85	14.5%
	Yes	500	85.5%
Are you aware that around the world there is a trend of teaching common people some simple techniques of bleeding control at the accident scene?	No	299	51.1%
	Yes	286	48.9%
If the answer is yes, what is the source of information	Social media	142	46.6%
	Print media	17	5.6%
	Friends and family	45	14.8%
	Healthcare-related posters in malls /healthcare facilities	101	33.1%
In your opinion, the use of tourniquets is	Not safe	22	3.8%
	Safe	326	55.7%
	Not sure	237	40.5%
Would you use a tourniquet in real life?	No	76	13.0%
	Yes	348	59.5%
	Not sure	161	27.5%
How would you rate your confidence level in regard to responding to medical emergencies?	Very uncomfortable	188	32.1%
	Uncomfortable	128	21.9%
	Somewhat comfortable	166	28.4%
	Comfortable	56	9.6%
	Very Comfortable	47	8.0%

**Figure 1:**
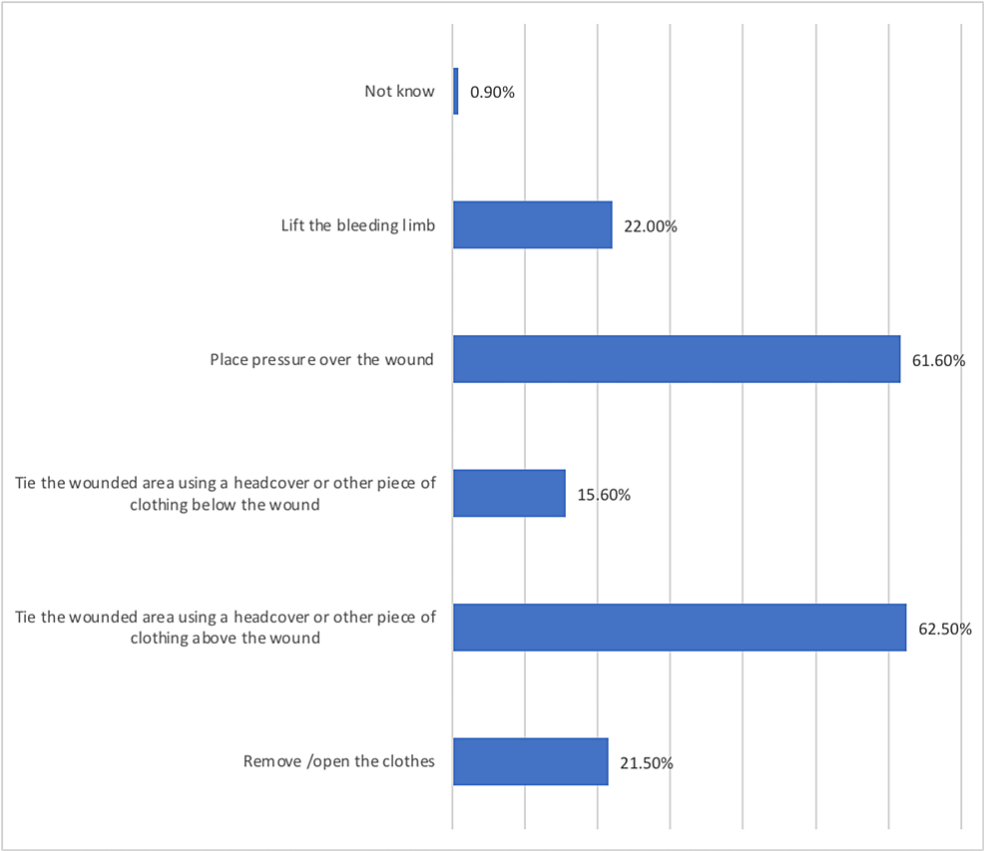
Ways to stop bleeding in the accident site according to the participants

Considering ways used by the participants to stop bleeding in the accident site, we found that 0.9% reported that they did not know any of these ways; however, placing pressure over the wound was the most popular ways used by the participants (61.6%) followed by tying the wounded area using headcover or other piece of clothing above the wound. Moreover, 22% would lift the bleeding limb, while 21.5% would start with removing or opening the clothes. Furthermore, 15.6% of the participants thought that tying the wounded area using headcover below the wound would stop the bleeding (Figure 1).

**Figure 2:**
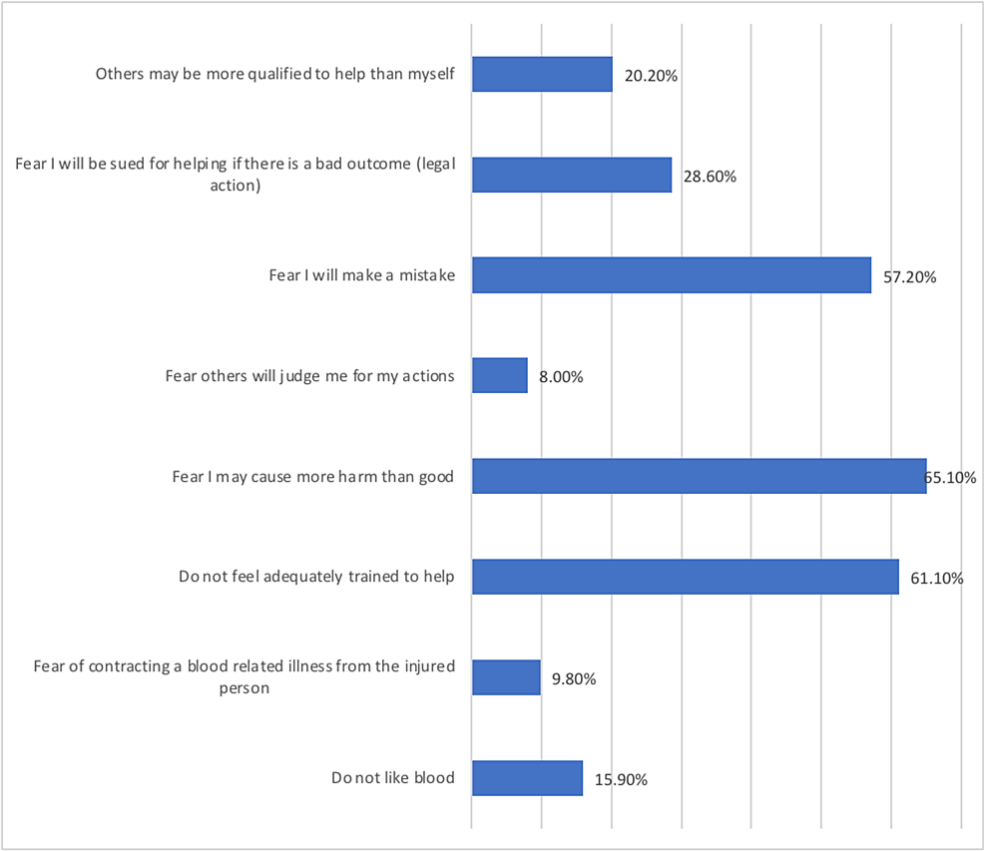
Reasons for not being comfortable helping in an emergency by applying a tourniquet to control bleeding

Fear of causing more harm to the patients was the main barrier in helping patients in an emergency by applying a tourniquet to control bleeding (65.1%) followed by feeling that they are not adequately trained to help (61.1%) and fear of making a mistake (57.2%). Moreover, fear of being sued for helping if there is a bad outcome was the barrier for 28.6% of the participants, followed by the thought that others may be more qualified to help them (20.2%) (Figure 2).

**Figure 3:**
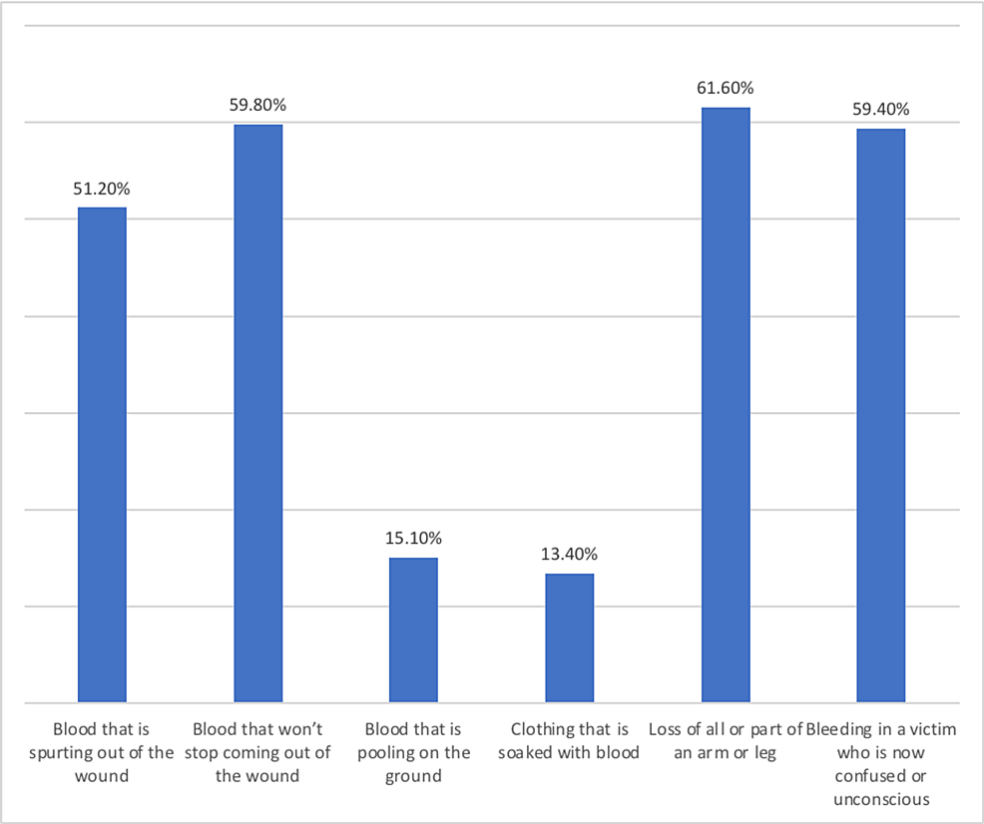
Examples of dangerous bleeding in participants' opinion

According to the participants, losing of all or part of an arm or leg is the most important example of dangerous bleeding (61.6%), followed by having unstoppable bleeding (59.8%), bleeding in victim who is confused or unconscious (59.4%), and blood that is spurting out of the wound (51.2%) (Figure 3).

Among the participants, 87.2% of them thought that tourniquets can be effective in stopping uncontrolled extremity bleeding, while 74.2% did not think that application of a tourniquet is complicated or difficult to learn. Moreover, 73.7% of the participants knew that tourniquets should not be delayed, 68.5% thought that tourniquets should be placed high and tight, and 49.9% thought that tourniquets should be placed for only one hour (Table 3).

**Table 3:** Awareness of the participants considering use of tourniquets

	False		True	
	N	%	N	%
Tourniquets can be an effective way to stop uncontrolled extremity bleeding	75	12.8%	510	87.2%
Tourniquet application is complicated and difficult to learn	434	74.2%	151	25.8%
Tourniquets should be delayed if possible	431	73.7%	154	26.3%
Tourniquets should be placed “high and tight”	184	31.5%	401	68.5%
Tourniquets can only be left in place for one hour	293	50.1%	292	49.9%

In general, we found that 40.2% of the participants have adequate knowledge considering controlling of bleeding in an emergency setting, while 59.8% had inadequate knowledge. Age, gender, nationality, income, profession, nor region of living had any effect on the level of knowledge of the participants, as shown in Table 4. Only educational level of the participants and having any previous first aid training were the factors affecting the level of knowledge. Higher level of education and having previous first aid training were associated with better knowledge significantly (p=0.001 and 0.012, respectively).

**Table 4:** Relationship between demographic factors and knowledge of the participants Asterisks denote the statistically significant results

		Knowledge				p-Value
		Inadequate		Adequate		
		N	%	N	%	
Age (years)	18-26	205	55.7%	163	44.3%	0.065
	27-36	70	64.2%	39	35.8%	
	37-46	34	75.6%	11	24.4%	
	47-56	27	64.3%	15	35.7%	
	>56	14	66.7%	7	33.3%	
Sex	Male	202	62.3%	122	37.7%	0.167
	Female	148	56.7%	113	43.3%	
Nationality	Saudi	315	59.7%	213	40.3%	0.799
	Non-Saudi	35	61.4%	22	38.6%	
Region	Central region	235	61.2%	149	38.8%	0.890
	Eastern region	28	56.0%	22	44.0%	
	Western region	56	58.9%	39	41.1%	
	Southern region	13	54.2%	11	45.8%	
	Northern region	18	56.3%	14	43.8%	
Level of education	High School	88	62.0%	54	38.0%	0.001*
	University	231	60.3%	152	39.7%	
	Postgraduate	30	54.5%	25	45.5%	
Income	<3000	176	59.3%	121	40.7%	0.738
	3000-5000	36	55.4%	29	44.6%	
	5000-7000	21	63.6%	12	36.4%	
	7000-10,000	26	66.7%	13	33.3%	
	10,000-15,000	35	62.5%	21	37.5%	
	15,000-20,000	24	63.2%	14	36.8%	
	20,000-30,000	13	46.4%	15	53.6%	
	>30,000	19	65.5%	10	34.5%	
Profession	Student	154	53.7%	133	46.3%	0.145
	Not working	20	62.5%	12	37.5%	
	Private sector employee	18	64.3%	10	35.7%	
	Teacher	29	69.0%	13	31.0%	
	Office work	72	63.7%	41	36.3%	
	Trader	10	71.4%	4	28.6%	
	Housewife	9	81.8%	2	18.2%	
	Other	38	65.5%	20	34.5%	
Do you have any previous first aid training?	No	279	62.7%	166	37.3%	0.012*
	Yes	71	50.7%	69	49.3%	

**Figure 4:**
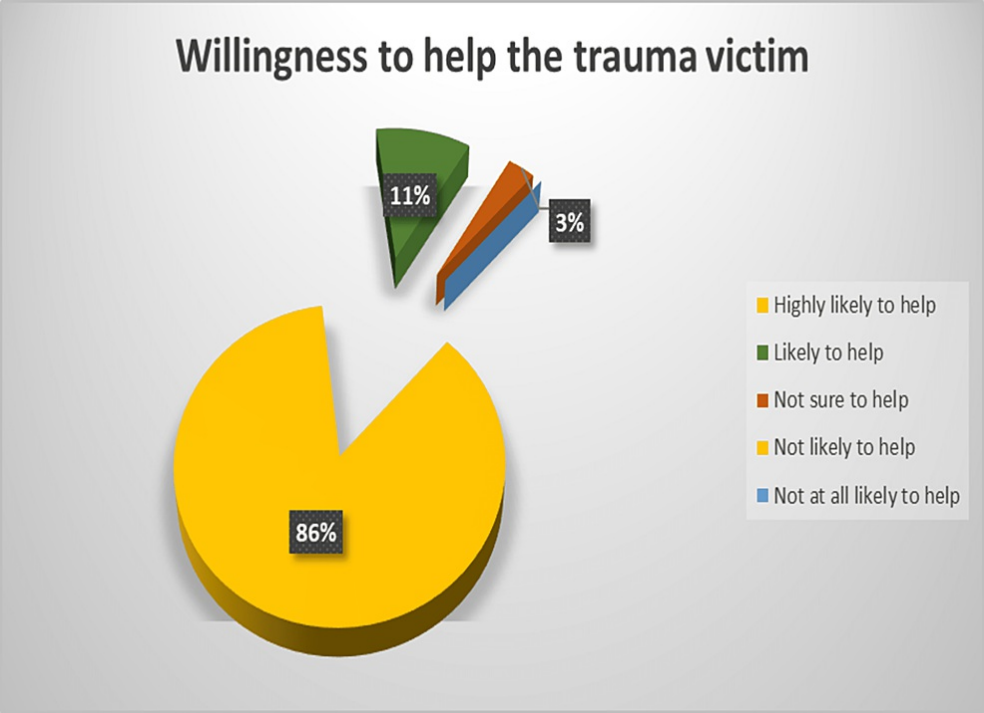
Willingness to help the trauma victim in real life.

Regarding the willingness to offer help to the trauma victims in real life, out of the 585 participants, 505 (86.3%) and 62 (10.6%) expressed that it is “highly likely” and “somewhat likely,” respectively, and only 18 (3.1%) were “unsure” of their response. No one registered that it was “unlikely” for him/her to respond in real-life situations (Figure 4).

Post-survey, 572 (87.8%) participants felt that the survey was helpful to them and that they would be willing to participate in healthcare-related surveys in future. There was no statistically significant difference in response between the various groups or the two genders.

## Discussion

First aid training is considered one of the important skills that should be gained by most of the people in our community where more than 50% of adults around the world reported having received some form of training at some point in their lives [8-10]. The high incidence of mortality among patients with traumatic bleeding after traffic accidents in Saudi Arabia [11] highlights the lack of the first aid training and response to disasters in the country, as indicated by our results that showed that only 23.9% of the participants reported having a previous medical training in bleeding control. This is similar to some studies conducted in Saudi Arabia among different populations including the study by Alyahya et al., which reported that only 26.4% of the teachers had received a first aid training before [12]., study by Halawani et al., which showed that the prevalence of university students who have first aid training was 26% [13], and the study by Mobarak et al., who reported that 13.6% of secondary students had first aid training before [14]. The same results also reported in other population than Saudi Arabia, including the study by Larsson et al., who reported that 25% of the respondents in Sweden population had participated at least once in some type of first aid training, 14% had participated in two or more training sessions, and 61% had no previous training [8]. In another study conducted among populations in Kuwait showed that 57% of the population had no prior exposure to any training in bleeding control [15].

Unintentional injuries are considered the leading cause of death among general population younger than 44 years in the USA [16]. In a Swedish study, the authors showed that 14% of the people reported presenting at the scene of road traffic accidents at least once in the five years prior to the study [8]. According to many studies, most of the bystanders at the crash site often concluded that first aid is unnecessary with a rate of immediate action being less than 35% of all victims as reported by the ambulance person arriving on the scene [17-20]. Moreover, some other studies reported that bystanders could have done more with cases of bleeding [8], with other studies showing that first aid training predicted actual utilization of these first aid skills [21]. These data combined are significant enough to indicate the importance of extensive first aid training of the general populations. In our study, we found that 40.2% of the participants had good and adequate knowledge of controlling of bleeding. Moreover, having previous first aid training has a significant impact on improvement of knowledge in bleeding control. In a previous study conducted in Kuwait, the authors reported a significant improvement in the knowledge of the participants in the type of response of the general public to basic traumas assessed, proving greater perceived abilities to handle a medical emergency by those who took part in it after application of awareness campaign (Stop the Bleed campaign) [15]. Another study conducted by McCarty et al. reported that prior form of first aid or bleeding control training increased the likelihood of successful completion of tasks in controlling bleeding [22]. The study showed that those who had first aid training had 2.12 higher odds of correct tourniquet application and those who had bleeding control training had 3.5 higher odds of correct applications compared with those with no training [22]. These results also reported by other studies that showed increase in confidence and knowledge in bleeding control after having training [23-26].

A tourniquet is a device that is placed around the bleeding arm or leg that works by squeezing large blood vessels, which helps in stopping blood loss [27]. Tourniquet can be made out of any available material including bandage, strip of cloth, or even a t-shirt [27]. In our study, most of the participants had good knowledge considering using a tourniquet and its importance. However, fear of causing more harm to the patients was the main barrier in helping patients in an emergency by applying a tourniquet to control bleeding (65.1%), followed by feeling that they are not adequately trained to help (61.1%) and fear of making a mistake (57.2%). In one of the previous studies, it was found that the fear of causing more harm to the patients or getting involved in medicolegal issues were the main barriers to help stop bleeding in the view of bystanders [15].

This study has certain limitations, which include dependence upon a self-reported questionnaire which has the potential to lead to personal bias. The responses to the questionnaire were recorded online via a Google sheet, and this methodology has the potential to lead to sampling bias by preferential recruitment of younger and educated participants with better hold over the newer technologies and evolving communication tools.

‏However, it is hoped that this pilot study would serve as a stimulus for initiation of large-scale studies and community-level practical training activities, thereby popularizing the idea of onsite bleeding control by bystanders.

Since the willingness to help the trauma victims is very high, bleeding control skills teaching activities at a community level are expected to give encouraging results in future.

Since social media and healthcare-related posters have been identified by the participants as the major source of information, these tools should be utilized for dissemination of the information. Social media would in fact serve as a very cost-effective medium as most of the material is already available on internet for free and requires only proper identification and broadcast of links. Translation into local Arabic language and creation of content would take the message to grassroots levels of the society.

## Conclusions

This study provides some insight into awareness, attitude, and willingness toward bleeding control at the accident site by bystanders living in Riyadh city. The awareness levels are generally poor, and there is strong need to devise effective strategies to improve this situation. The possible strategies include incorporation of bleeding control skills in school curriculum and conduct of simulation activities such as “Stop the Bleed campaign” at shopping malls, academic institutions, and offices. Judicious use of social media and modern communication modalities can be helpful in this regard. The efforts made in this direction would alleviate the fear and hesitancy, thereby converting passive bystanders into an active force, and that positive transformation would in coming times potentially decrease the magnitude of mortalities associated with trauma.

## Appendices

1. https://youtube.com/watch?v=mhBe7Q6mH3U&feature=share

2. https://emergency.utah.edu/wp-content/uploads/sites/28/2021/03/Stop-the-Bleed-Booklet-Final.pdf

## References

[1] Toroyan T, Peden MM, Iaych K (2013). WHO launches second global status report on road safety. Inj Prev.

[2] World Health Organization (2019). World Health Statistics 2019: Monitoring Health for the SDGs, Sustainable Development Goals. https://injuryprevention.bmj.com/content/19/2/150.

[3] Catmull SP, Ashurst J V (2022). Autotransfusion. StatPearls [Internet].

[4] Stensballe J, Henriksen HH, Johansson PI (2017). Early haemorrhage control and management of trauma-induced coagulopathy: the importance of goal-directed therapy. Curr Opin Crit Care.

[5] Davoodabadi A, Abdorrahim Kashi E, Mohammadzadeh M (2021). Predicting factors and incidence of preventable trauma induced mortality. Ann Med Surg (Lond).

[6] Ramachandra G, Ramana Rao GV, Tetali S (2021). Active bleeding control pilot program in India: simulation training of the community to stop the bleed and save lives from Road Traffic Injuries. Clin Epidemiol Glob Heal.

[7] (2022). Population in Riyadh region by gender, age group, and nationality. https://www.stats.gov.sa/en/5721.

[8] Larsson EM, Mártensson NL, Alexanderson KA (2002). First-aid training and bystander actions at traffic crashes--a population study. Prehosp Disaster Med.

[9] Clark MJ, Enraght-Moony E, Balanda KP, Lynch M, Tighe T, FitzGerald G (2002). Knowledge of the national emergency telephone number and prevalence and characteristics of those trained in CPR in Queensland: baseline information for targeted training interventions. Resuscitation.

[10] Swor R, Compton S, Farr L, Kokko S, Vining F, Pascual R, Jackson RE (2003). Perceived self-efficacy in performing and willingness to learn cardiopulmonary resuscitation in an elderly population in a suburban community. Am J Crit Care.

[11] Mansuri FA, Al-Zalabani AH, Zalat MM, Qabshawi RI (2015). Road safety and road traffic accidents in Saudi Arabia. A systematic review of existing evidence. Saudi Med J.

[12] AlYahya IA, Almohsen HA, AlSaleem IA (2019). Assessment of knowledge, attitude, and practice about first aid among male school teachers and administrators in Riyadh, Saudi Arabia. J Family Med Prim Care.

[13] Halawani LM, Alghamdy SD, Alwazae MM, Alkhayal WA (2019). Knowledge and attitude of Saudi female university students about first aid skills. J Family Community Med.

[14] Mobarak AS, Afifi RM, Qulali A (2015). First aid knowledge and attitude of secondary school students in Saudi Arabia.. Health (Irvine Calif).

[15] AlSabah S, Al Haddad E, AlSaleh F (2018). Stop the bleed campaign: a qualitative study from our experience from the middle east. Ann Med Surg (Lond).

[16] World Health Organization (1999). Injury: A Leading Cause of the Global Burden of Disease. Published online.

[17] Lund I, Skulberg A (1976). Cardiopulmonary resuscitation by lay people. Lancet.

[18] Jackson RE, Swor RA (1997). Who gets bystander cardiopulmonary resuscitation in a witnessed arrest?. Acad Emerg Med.

[19] Axelsson Å, Herlitz J, Karlsson T (1998). Factors surrounding cardiopulmonary resuscitation influencing bystanders’ psychological reactions. Resuscitation.

[20] Murphy RJ, Luepker RV, Jacobs DR Jr, Gillum RF, Folsom AR, Blackburn H (1984). Citizen cardiopulmonary resuscitation training and use in a metropolitan area: the Minnesota Heart Survey. Am J Public Health.

[21] Kano M, Siegel JM, Bourque LB (2005). First-aid training and capabilities of the lay public: a potential alternative source of emergency medical assistance following a natural disaster. Disasters.

[22] McCarty JC, Caterson EJ, Chaudhary MA (2019). Can they stop the bleed? Evaluation of tourniquet application by individuals with varying levels of prior self-reported training. Injury.

[23] Kelley K, Martinson J, Henry S, Scalea T, Park H (2022). Have students used techniques to stop the bleed?. Am Surg.

[24] Goralnick E, Chaudhary MA, McCarty JC (2018). Effectiveness of instructional interventions for hemorrhage control readiness for laypersons in the Public Access and Tourniquet Training Study (PATTS): a randomized clinical trial. JAMA Surg.

[25] Latuska KM, Graf RL, Zwislewski A, Meyer LK, Nanassy AD (2019). Stop the bleed training improves knowledge, skills, and confidence among school nurses. J Contin Educ Nurs.

[26] Andrade EG, Hayes JM, Punch LJ (2020). Stop the bleed: the impact of trauma first aid kits on post-training confidence among community members and medical professionals. Am J Surg.

[27] Galante JM (2017). Using tourniquets to stop bleeding. JAMA.

